# Fungicide Exposure in Honey Bee Hives Varies By Time, Worker Role, and Proximity to Orchards in Spring

**DOI:** 10.1093/jee/toad008

**Published:** 2023-01-27

**Authors:** Jacquelyn A Perkins, Kyungmin Kim, Larry J Gut, George W Sundin, Julianna K Wilson

**Affiliations:** Department of Entomology, Michigan State University, East Lansing, MI, USA; Department of Plant, Soil and Microbial Sciences, Michigan State University, East Lansing, MI, USA; Department of Entomology, Michigan State University, East Lansing, MI, USA; Department of Plant, Soil and Microbial Sciences, Michigan State University, East Lansing, MI, USA; Department of Entomology, Michigan State University, East Lansing, MI, USA

**Keywords:** pollination, environmental impact, pesticide, integrated pest management, cherry

## Abstract

Fungicides are commonly applied to prevent diseases in eastern North American cherry orchards at the same time that honey bees (*Apis mellifera* L. (Hymenoptera: Apidae)) are rented for pollination services. Fungicide exposure in honey bees can cause negative health effects. To measure fungicide exposure, we sampled commercial honey bee colonies during orchard bloom at two commercial tart cherry orchards and one holding yard in northern Michigan over two seasons. Nurse bees, foragers, larvae, pollen, bee bread, and wax were screened for captan, chlorothalonil, and thiophanate-methyl. We also looked at the composition of pollens collected by foragers during spring bloom. We found differences in fungicide residue levels between nurse bees and foragers, with higher captan levels in nurse bees. We also found that residue levels of chlorothalonil in workers were significantly increased during tart cherry bloom, and that nurse bees from hives adjacent to orchards had significantly higher chlorothalonil residues than nurse bees from hives kept in a holding yard. Our results suggest that fungicide exposure of individual honey bees depends greatly on hive location in relation to mass-flowering crops, and worker role (life stage) at the time of collection. In some pollen samples, captan and chlorothalonil were detected at levels known to cause negative health effects for honey bees. This study increases our understanding of exposure risk for bees under current bloom time orchard management in this region. Further research is needed to balance crop disease management requirements with necessary pollination services and long-term pollinator health.

Managed honey bee (*Apis mellifera* L.) colonies contribute more than $15 billion in value to the United States economy each year in pollination services (Holdren 2015). Temperate fruit growers typically rent hives from beekeepers to increase yield and fruit quality (Delaplane and Mayer 2000). Pesticide exposure is an important concern for honey bees that are used in commercial pollination and is one of the factors implicated in annual colony losses (Johnson 2010, Berenbaum 2016, Colwell et al. 2017). Foraging bees may be exposed to pesticides through direct spray, contact with treated crops, drift onto nearby floral resources, or contaminated surface water (Sanchez-Bayo and Goka 2014, Long and Krupke 2016, McCune et al. 2021). Nurse bees inside the hive can be exposed to residues when they interact with contaminated foragers, as they process contaminated pollen and nectar, and through contact with contaminated bees wax (Sponsler and Johnson 2016, Rondeau and Raine 2022).

Tree fruit growers use a variety of pesticides to protect their crops, including fungicides to prevent bloom-time diseases (USDA NASS 2017). In Eastern North America, emerging green tissue and flowers on trees in the spring are highly vulnerable to infection by fungal and bacterial pathogens. Key fungal diseases targeted in spring include cherry leaf spot (*Blumeriella jaapii* (Rehm), Helotiales: Dermateaceae) and American brown rot (*Monilinia fructicola* (G. Winter) Honey, Helotiales: Schlerotiniaceae) in tart cherries, and apple scab (*Venturia inaequalis* (Cooke) G. Winter, Pleosporales: Venturiaceae) in apples (Jones and Sutton 1984). If uncontrolled, cherry leaf spot and apple scab can lead to premature defoliation and direct crop damage. American brown rot can kill flowers and cause subsequent fruit loss. Growers typically begin applying fungicides right before bloom and rotate spray programs between broad-spectrum fungicides and more targeted materials to minimize fungicide resistance (Proffer et al. 2006, Lesniak et al. 2011, Outwater et al. 2019, Wise 2022). While insecticide labels often place legal restrictions on applications during bloom to protect pollinators, most fungicides are not restricted for use during crop bloom at this time.

From pesticide residue surveys of hives kept by migratory beekeepers, we know that fungicide residues are commonly found in pollen (Mullin et al. 2010, Sanchez-Bayo and Goka 2014, Rondeau and Raine 2022). Friedle et al. (2021) found that fungicides accounted for 51% of pesticide contaminants in bee-collected pollen from hives near fruit-growing regions of Southern Germany. Chlorothalonil and captan are among the active ingredients used in Michigan in early spring to protect orchards from disease at a time that overlaps with bloom and pollinator activity (Wise 2022). Both fungicides have been detected in honey bee hives from previous studies, and captan is one of 18 fungicides that have been detected in all five honey bee matrices (honey, pollen, bee bread, wax, and honey bees) (Rondeau and Raine 2022). Although both chlorothalonil and captan have low acute toxicity to honey bees, both chemicals are currently listed in category II of the UC IPM Bee Precaution Pesticide Ratings, meaning that they are considered by this organization to be toxic to honey bees, due to potential impacts on developing honey bee brood and other sub lethal effects (https://www2.ipm.ucanr.edu/beeprecaution/). Mussen et al. (2004) found detrimental effects on honey bee development when larvae were exposed to captan. Recent laboratory studies have linked levels of chlorothalonil to sub-lethal health effects in honey bees, including increased larval mortality, increased *Nosema apis* (Zander) (Nosematidae) infection rates, and disruptions to the gut microbiome (Pettis et al. 2013, Zhu et al. 2014, Degrandi-Hoffman et al. 2015, Kakumanu et al. 2016). High levels of chlorothalonil have been detected in hives in fresh-collected pollen, beebread, and bees wax (Rondeau and Raine 2022).

Here we quantified real world fungicide exposure of honey bees contracted for pollination of *Prunus cerasus* L. (Rosaceae) cv. Montmorency tart cherries in northern Michigan, where two-thirds of all the Montmorency cherries are grown in the US (https://www.choosecherries.com/tart-cherries-101/). Quantifying honey bee fungicide exposure in this highly specialized crop and unique growing region is an important step to understanding how honey bees may be impacted by these pollination contracts. Many beekeepers in the US are migratory, meaning they move their bees from crop to crop to provide pollination (Colwell et al. 2017). Many of the hives that provide pollination for Michigan cherry orchards are first providing pollination services to almond orchards in California, and after cherry bloom are moved again for cucumber pollination, or to honey production yards (D. Nesky, pers. comm.). Understanding the fungicide residues entering hives during the short period of cherry bloom in Michigan provides a meaningful context for how orchard management strategies may be impacting bees brought in to provide pollination for this industry. We screened honey bee colonies for residues of three broad spectrum fungicides (chlorothalonil, captan, and thiophanate methyl) and compared fungicide exposure levels in hives rented for tart cherry pollination with hives kept in a holding yard (i.e., not rented for crop pollination and placed in a semi-natural habitat rather than an orchard) within the same region. The specific objectives were 1) to quantify field-level fungicide residues in nurse bees, foragers, larvae, pollen, bee bread, and wax, 2) to determine if worker role influences fungicide exposure levels, and 3) to characterize the pollens collected by honey bees around tart cherry bloom as they relate to fungicide residues.

## Materials and Methods

### Study Area and Design

Eight honey bee hives were sampled at each of three sites per year near Traverse City, Michigan during the springs of 2016 and 2017 (*n* = 48 hives) (Supp Table 1 [online only]). Before bloom each year, palleted hives in sets of four were placed by the beekeeper either next to commercial tart cherry orchards within range of other commercial fruit production, or in a holding yard surrounded by semi-natural areas not directly adjacent to orchards. After bloom, orchard hives were moved to the holding yard. We used the USDA NASS Cropland Data Layer to quantify land use in ArcGIS within a 2 km radius around each set of hives, selected based on an expected median foraging radius of 1.7 km in temperate forested regions (Winston 1991). Land use was consolidated into five categories: 1) orchard or vineyard, 2) other cultivated crops, 3) low density development, 4) semi-natural or noncultivated habitats (primarily forest), and 5) open water. Land use around the orchard sites was comprised of 21% orchard or vineyard, 7% other cultivated crops, 7.5% developed, 62% semi-natural, and 2.6% open water. Different holding yards, about 7 km apart, had to be used in 2016 versus 2017 due to beekeeper logistics, but the surrounding composition of land use was very similar between the two sites with 2.7 and 0.5% orchard or vineyard, 2.4 and 1.4% other cultivated crops, 8.6 and 5.6% developed, 86 and 92% semi-natural, and 0.04 and 0.01% open water, respectively. All hives used were owned and maintained by the same commercial beekeeper, who used consistent management practices to maximize bee health and productivity amongst all hives in the study. All tart cherry orchards to which hives were deployed were owned and managed by the same grower.

Though we centered this study on tart cherry bloom, within foraging range of the hives were also commercial sweet cherry (*Prunus avium* L. (Rosaceae)) and apple (*Malus domestica* Borkh. (Rosaceae)) orchards, as well as wild *Prunus* spp. and other deciduous trees and woodland spring blooming forbs. Sweet cherry bloom can be just finishing as tart cherry bloom begins; in some years, these bloom events can overlap by a few days. The earliest apple cultivars usually begin to bloom one to two weeks after the end of tart cherry bloom. Since residues from pesticide applications made in nearby orchards could also be picked up by honey bees intended for tart cherry pollination, we obtained spray records from our grower cooperator for both cherry and apple orchards adjacent to the studied hives. According to these records, nearby tart cherry orchards received two fungicide applications before bloom, and two to four applications during bloom. Fungicide active ingredients applied included chlorothalonil, thiophanate-methyl, fenbuconazole, fluopyram, and trifloxystrobin. In apple, between 4 and 6 fungicide applications were made leading up to and during crop bloom, which included the fungicide active ingredients of captan, trifloxystrobin, and mancozeb. We focused our screening efforts on chlorothalonil, captan, and thiophanate-methyl, all commonly used broad-spectrum fungicides that could be detected using the same extraction methods.

### Sample Collection

Sample collections were made once from each hive at timings corresponding to tart cherry *prebloom*, *peak bloom*, and *postbloom*, unless otherwise noted. Only queen-right colonies were used for sample collection; hives were deemed ‘queen-right’ if either the queen and/or fresh brood were observed.

To sample for nurse bees, brood frames were lifted out of the hive, gently shaken to disperse likely foragers, and then 10 g of bees were scooped directly from the frame into a zip-top bag. Nurse bees were collected in both years, but there is no postbloom sample for 2016 due to black bear damage to hives. Foragers were collected as they returned to the hive when temperatures were above 10°C and wind speeds less than 16 kph using insect vacuums (Insect Vacuum 18 Volt Cordless, BioQuip; Rancho Dominguez, CA) for 1 minute at the hive entrance. Foragers were only collected in 2017. Nurse and forager samples were placed on ice for transport, then frozen in the laboratory at −20°C until processing.

In 2017, pollen traps (10-Frame Superior Pollen Trap, Mann Lake; Hackensack, MN) were installed under each of two hives at both the holding yard and one orchard site (*n* = 4) by the beekeeper before the first sampling round. Traps were set to collect pollen during a 5-d period during peak bloom. Pollen that accumulated in the traps without the traps being set was collected at the prebloom, early bloom (immediately before the 5-d period when traps were set), and postbloom timings. Pollen samples were transferred to a zip-top bag, placed on ice, and frozen at −20°C until processing.

In 2016, a new drone frame (Mann Lake; Hackensack, MN) was placed in each hive before the study period and collected at postbloom to collect freshly drawn wax for analysis. In 2017, one existing brood frame from each hive was removed between the bloom and postbloom sampling periods to collect wax, bee bread, and larvae. Frames were placed individually into large plastic bags, transported on ice, and frozen at −20°C until processing. Wax was scraped from each frame into glass vials. Bee bread and capped brood were collected from frames using sterilized tweezers. Similar aged larvae were sampled by opening a capped cell and selecting only those that had not yet begun to pupate (approx. 4–5th instars).

### Sample Preparation and Fungicide Extraction

All samples (adult bees, larvae, pollen, bee bread, and wax) were screened for chlorothalonil, captan, and thiophanate-methyl using the QuEChERS method (Wiest et al. 2011). Samples (10 g) were combined with sodium chloride (1 g), magnesium sulfate (4 g), and enough high-performance liquid chromatography grade dichloromethane (British Drug Houses, Poole, United Kingdom) to completely cover the sample in glass vials (118.3 ml). Vials were capped, inverted twice, agitated for 30 min, then stored at 23°C for 2 wk for passive extraction. After extraction, each sample was muddled and topped off with additional dichloromethane as needed to cover samples in liquid.

Each sample was decanted and filtered (Whatman #1 filter paper Sigma-Aldrich, St. Louis, MO) into a clean glass vial (118.3 ml) with sodium sulfate (4 g) and left uncapped in a fume hood until all liquid had evaporated (~48 hr). Once dry, acetonitrile (2 ml) was added to each vial, gently swirled, and sonicated for 1 min. The liquid was filtered through a Polytetrafluorethylene (PTFE) membrane syringe filter (0.45 μm pore size, Scientific Equipment of Houston; Navasota, TX) into a small glass vial (2 ml) that was capped, labeled, and placed in the refrigerator at 2.2°C until analysis. Gas chromatography analysis was conducted by the Michigan State University Pesticide Analytical Lab using standard protocols for gas chromatography-mass spectrometry (GC-MS). Limit of Quantification (LOQ) and Limit of Detection (LOD) data for each fungicide are listed in Supp Table 2 (online only) of supplemental materials. Controls were spiked at the level of LOQ for each individual pesticide with an expected efficiency of recovery between 50–100%. For samples in which no residues were detected above the LOQ, we applied a value equal to the LOD for the selected compound in statistical analyses, because we cannot be sure these are true zeros based on the limitations of our analytical methods.

### Pollen Identification

To determine the identification of pollens collected during the study, a mortar and pestle were used to grind pollen (150 g), which were then dried in paper bags for 24 hr in a drying oven at 60°C. After drying, samples were shipped to the U.S. Geological Survey (USGS), Leetown Science Center, Kearneysville, WV where DNA was extracted (Doyle 1991). PCR amplification, sequencing, and bioinformatics analysis using the lowest-common ancestor approach were applied (Cornman et al. 2015). Raw counts of reads were converted to proportions within samples to compare collection composition. Taxa that could be identified to a genus and comprised more than 5% of any of the samples were included in the analyses described below.

### Statistical Analysis

To explore differences in residue exposure associated with worker role (nurse vs. forager), hive location (holding yard vs. orchard), and sample period (prebloom, bloom, postbloom of tart cherry), chlorothalonil and captan levels were analyzed separately using a repeated measures approach generated using PROC MIXED procedure of SAS version 9.4 (SAS Institute Inc., Cary, NC). The model included the fixed effects of site, sample type, period, and their combinations, and a random effect of hive nested within site. As a subject for repeated measurements, the combination of type and hive nested with site was used (Milliken and Johnson 2009). The best variance-covariance structure was selected by comparing the Akaike Information Criterion (AIC) and Bayesian Information Criterion (BIC). Compound symmetry with a heterogenous variance model was selected as the final model for both pesticide types. When the interaction between two factors was significant, slicing (i.e., simple effect test of the interactions) was conducted to examine the interaction effects. All results were interpreted as statistically significant at *p* < 0.05 level. Assumptions of normality were evaluated by checking the probability plot of the residuals before the main analysis. For chlorothalonil, data were natural-log transformed to correct for normal distribution, however, back-transformed 95% confidence intervals are reported as results.

To explore environmental correlations between the kinds of pollen collected and the fungicide residues being picked up by hives in our study, we applied a Principal Component Analysis (PCA) using the ‘prcomp’ function in the ‘stats’ package (Lüdecke et al. 2020). Our multivariate dataset included the centered-log-ratio-transformed raw counts of the 12 most abundant pollen genera collected by 4 honey bee hives at 4 different time periods related to tart cherry bloom (prebloom, early bloom, late bloom, and postbloom) and the residues of the two fungicides that were detected (chlorothalonil and captan) measured in mg kg^−1^ obtained from the same samples in 2017.

A scree plot of the eigenvalues was used to determine the number of significant Principal Component (PC) axes to retain for subsequent analysis using the Kaiser-Guttman criterion (Legendre and Legendre 1998) (Supp Fig. 1 [online only]). We also generated bivariate plots of pairs of significant PCs (i.e., based on their scores, eigenvectors) and used the ‘envfit’ function in R’s ‘vegan’ package v. 2.6 to include factor loading vectors (i.e., correlation coefficients) of the two fungicides to the plots (Oksanen et al. 2019). This allowed us to describe and investigate how fungicide applications coincided with hives and their locations as well as changing bloom periods.

Principal component regressions were used to provide quantitative support for our visual (qualitative) interpretations of the bivariate ordination of PC1 and PC2 using the ‘lm’ function in the ‘stats’ package (R Core Team 2020), fitting an ordinary least squares regression in each of two different linear models. For the first model, raw PC1 scores comprised the dependent variable and fungicide residues (continuous covariate) comprised the independent variable. For the second model, raw PC2 scores comprised the dependent variable and bloom period (categorical factor) comprised the independent variable. Estimating these two models allowed us to understand how PC scores correlated with fungicide concentrations as well as different bloom periods, independently – numerically similar to estimates of factor loadings obtained from PCA. Before interpreting final parameter estimates of each model, we also evaluated whether or not the data adequately satisfied the assumptions of a general linear model (e.g., residual normality, independent observations, and homogeneity of group variance), applying various functions within R’s ‘performance’ package v. 0.4.5, including the ‘check_normality’ (Shapiro-Wilk test), ‘check_autocorrelation’ (Durbin-Watson test), and ‘check_heteroscedasticity’ functions on model residuals.

## Results

### Fungicide Residues

We screened for captan, chlorothalonil, and thiophanate-methyl residues in samples of nurse bees (*n* = 120), foragers (*n* = 72), larvae (*n* = 24), pollen (*n* = 15), bee bread (*n* = 24), and wax (*n* = 48) (Table 1). Thiophanate-methyl was not detected in any of the samples. Chlorothalonil and captan were detected above the LOQ in 35.1 and 47.3% of all the samples, respectively. Detection frequency, average residues, and max mg kg^−1^ associated with hive location, sample type, bloom period, and year are specified for all years, sites, and sampling periods in Table 1.

**Table 1.** Mean (±SEM) and maximum (Max.) residue levels (mg kg^−1^) of chlorothalonil and captan detected for each sample type by year, with respect to tart cherry bloom (period) at three sites. A ‘–’ indicates that no residue was detected above the LOQ for a given set of samples. For samples in which no residues were detected above the LOQ, we applied a value equal to the LOD for the selected compound

Type	Year	Period	Collection Date	Site	Chlorothalonil			Captan		
					Samples > LOQ	Mean ± SEM (mg kg^−1^)	Max. (mg kg^−1^)	Samples > LOQ	Mean ± SEM (mg kg^−1^)	Max. (mg kg^−1^)
Nurse (*n*= 120)	2016	Prebloom	9 May 16	Holding yard	0/8	–	–	1/8	2.25 ± 2.24	17.91
			10 May 16	Orchard-p	2/8	0.12 ± 0.08	0.61	0/8	–	–
			10 May 16	Orchard-v	3/8	0.11 ± 0.06	0.43	0/8	–	–
		Bloom	24 May 16	Holding yard	3/8	0.08 ± 0.06	0.47	2/8	1.31 ± 0.89	6.51
			23 May 16	Orchard-p	0/8	–	–	0/8	–	–
			23 May 16	Orchard-v	0/8	–	–	1/8	3.03 ± 3.03	24.22
	2017	Pre-bloom	9 May 17	Holding yard	0/8	–	–	8/8	24.84 ± 3.05	36.10
			10 May 17	Orchard-p	3/8	0.12 ± 0.07	0.54	8/8	25.21 ± 2.97	35.80
			10 May 17	Orchard-v	0/8	–	–	8/8	16.29 ± 1.85	21.90
		Bloom	16 May 17	Holding yard	0/8	–	–	8/8	22.00 ± 4.18	47.40
			16 May 17	Orchard-p	8/8	1.42 ± 0.24	2.48	7/8	4.48 ± 0.87	7.94
			15 May 17	Orchard-v	8/8	0.72 ± 0.13	1.42	5/8	10.71 ± 3.94	31.80
		Postbloom	31 May 17	Holding yard	0/8	–	–	8/8	28.58 ± 4.38	40.00
			31 May 17	Orchard-p	0/8	–	–	8/8	36.21 ± 6.71	75.30
			1 June 17	Orchard-v	2/8	0.12 ± 0.08	0.57	7/8	17.30 ± 3.65	32.40
Forager (*n* = 72)	2017	Prebloom	9 May 17	Holding yard	0/8	–	–	3/8	1.65 ± 1.09	8.85
			10 May 17	Orchard-p	2/8	0.16 ± 0.13	1.04	5/8	2.25 ± 0.68	4.31
			10 May 17	Orchard-v	0/8	–	–	5/8	11.82 ± 3.80	23.90
		Bloom	19 May 17	Holding yard	0/8	–	–	2/8	1.58 ± 1.17	9.22
			15 May 17	Orchard-p	8/8	2.37 ± 0.81	7.17	7/8	21.77 ± 8.59	62.00
			18 May 17	Orchard-v	8/8	17.99 ± 8.73	73.20	0/8	–	–
		Postbloom	1 June 17	Holding yard	0/8	–	–	0/8	–	–
			1 June 17	Orchard-p	0/8	–	–	0/8	–	–
			1 June 17	Orchard-v	1/8	0.07 ± 0.07	0.53	0/8	–	–
Larvae (*n* = 24)	2017	Bloom	19 May 17	Holding yard	0/8	–	–	0/8	–	–
			19 May 17	Orchard-p	0/8	–	–	0/8	–	–
			18 May 17	Orchard-v	0/8	–	–	0/8	–	–
Pollen (*n* = 15)	2017	Prebloom	9 May 17	Holding yard	0/2	–	–	2/2	4.19 ± 1.94	6.13
			10 May 17	Orchard-p	1/1	n/a	0.24	1/1	n/a	4.85
		Early bloom	16 May 17	Holding yard	2/2	0.62 ± 0.38	1.00	2/2	13.10 ± 1.60	14.70
			15 May 17	Orchard-p	2/2	6.26 ± 1.40	7.65	2/2	7.32 ± 0.43	7.75
		Late bloom	19 May 17	Holding yard	2/2	1.87 ± 0.29	2.15	2/2	21.90 ± 2.50	24.40
			18 May 17	Orchard-p	2/2	14.94 ± 6.16	21.10	2/2	3.59 ± 0.17	3.76
		Postbloom	31 May 17	Holding yard	2/2	2.32 ± 1.51	3.83	2/2	9.43 ± 2.88	12.30
			31 May 17	Orchard-p	2/2	6.31 ± 0.11	6.41	2/2	11.49 ± 5.11	16.60
Bee bread (*n* = 24)	2017	Bloom	19 May 17	Holding yard	2/8	0.13 ± 0.10	0.85	8/8	19.50 ± 2.52	30.90
			19 May 17	Orchard-p	8/8	3.92 ± 0.97	10.30	8/8	7.81 ± 2.24	20.30
			18 May 17	Orchard-v	8/8	3.89 ± 0.67	7.84	8/8	1.99 ± 0.58	5.15
Wax (*n* = 48)	2016	Bloom	24 May 16	Holding yard	1/8	0.01 ± 0.01	0.07	0/8	–	–
			23 May 16	Orchard-p	6/8	0.22 ± 0.05	0.43	0/8	–	–
			23 May 16	Orchard-v	7/8	0.15 ± 0.04	0.31	0/8	–	–
	2017	Bloom	19 May 17	Holding yard	0/8	–	–	0/8	–	–
			19 May 17	Orchard-p	1/8	0.02 ± 0.02	0.16	0/8	–	–
			18 May 17	Orchard-v	4/8	0.24 ± 0.10	0.67	0/8	–	–

#### Larvae and Workers

**Fig. 1.**
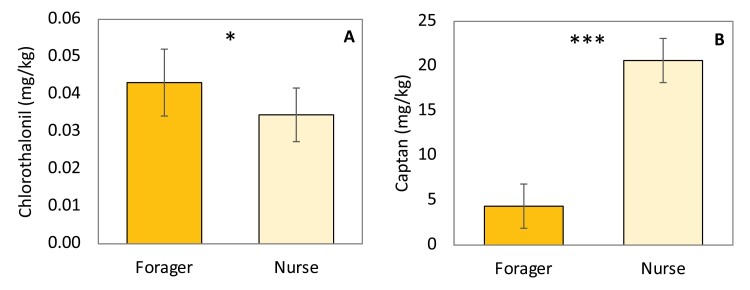
Effect of worker role on residue levels. Average residues of chlorothalonil (A) and captan (B) detected in foragers and nurse bees in 2017 (* indicates α < 0.05, *** indicates α < 0.0001).

No residues were detected in any of the larvae collected (Table 1). In 2017, all chlorothalonil detections in both nurse bees and foragers occurred in samples from hives next to orchards, with no chlorothalonil detected in any worker bees from the holding yard. Captan was detected in both nurse bees and foragers from hives at all three locations, but captan detections in foragers were confined to the prebloom through bloom periods (Table 1). Foragers showed a higher level of chlorothalonil residues compared to nurse bees (*F* = 4.82, df = 1,24.3, *p* = 0.0380) (Fig. 1A). However, nurse bees had significantly higher captan residues than foragers (*F* = 88.35, df = 1,43.2, *p* < 0.0001). (Fig. 1B).

**Fig. 2.**
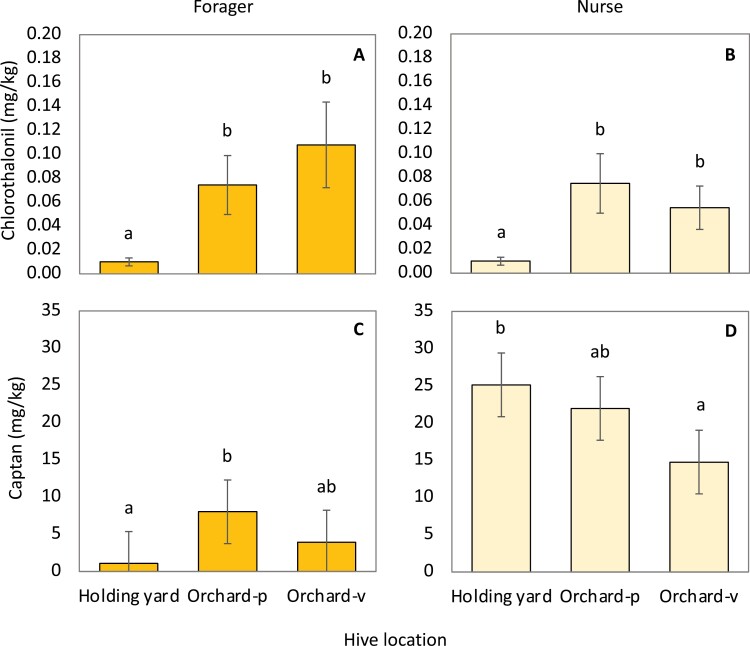
Hive location effects on residue levels in worker bees. Average residues of chlorothalonil or captan detected in foragers (A, C) and nurse bees (B, D) in 2017. Means with different lowercase letters are statistically significant at α = 0.05.

Both nurse bees and foragers from orchard sites had significantly higher chlorothalonil residues when compared to nurse bees and foragers collected from holding yards (Fig. 2A and B) (Table 2). Captan residues in foragers were significantly higher at orchard-p compared to the foragers from the holding yard, however, captan residues in foragers from Orchard-v were not statistically different than those from the holding yard (Fig. 2C) (Table 2). Captan residues in nurse bees from the holding yard were significantly higher than residues in nurse bees from Orchard-v, but there was no difference between the holding yard and Orchard-p (Fig. 2D) (Table 2).

**Table 2.** Least squares means comparisons of location and temporal effects of fungicide residues in workers honey bees collected from hives

Fungicide type	Worker type	Means comparison	df	*t*	*p*
Chlorothalonil	Forager	Holding yard vs. orchard-p	29	−7.19	<0.0001***
		Holding yard vs. orchard-v	29	−8.53	<0.0001***
		Orchard-p vs. orchard-v	29	−1.34	0.1913
	Nurse	Holding yard vs. orchard-p	29	−7.23	<0.0001***
		Holding yard vs. orchard-v	29	−6.10	<0.0001***
		Orchard-p vs. orchard-v	29	1.13	0.2664
Captan	Forager	Holding yard vs. orchard-p	43.2	−2.31	0.0256*
		Holding yard vs. orchard-v	43.2	−0.96	0.3441
		Orchard-p vs. orchard-v	43.2	1.35	0.1825
	Nurse	Holding yard vs. orchard-p	43.2	1.06	0.2963
		Holding yard vs. orchard-v	43.2	3.46	0.0012*
		Orchard-p vs. orchard-v	43.2	2.40	0.0207*
Chlorothalonil	Forager	Prebloom vs. bloom	53.6	−12.75	<0.0001***
		Prebloom vs. postbloom	59.7	0.55	0.5829
		Bloom vs. postbloom	55	16.29	<0.0001***
	Nurse	Prebloom vs. bloom	53.6	−9.26	<0.0001***
		Prebloom vs. postbloom	59.7	0.30	0.7649
		Bloom vs. postbloom	55	11.70	<0.0001***
Captan	Forager	Prebloom vs. bloom	55.4	−0.91	0.3654
		Prebloom vs. postbloom	59.9	2.11	0.0389*
		Bloom vs. postbloom	62	2.47	0.0162*
	Nurse	Prebloom vs. bloom	55.4	3.48	0.0010*
		Prebloom vs. postbloom	59.9	−2.12	0.0379*
		Bloom vs. postbloom	62	−476	<0.0001***

***Indicates α < 0.001, *indicates α < 0.05.

**Fig. 3.**
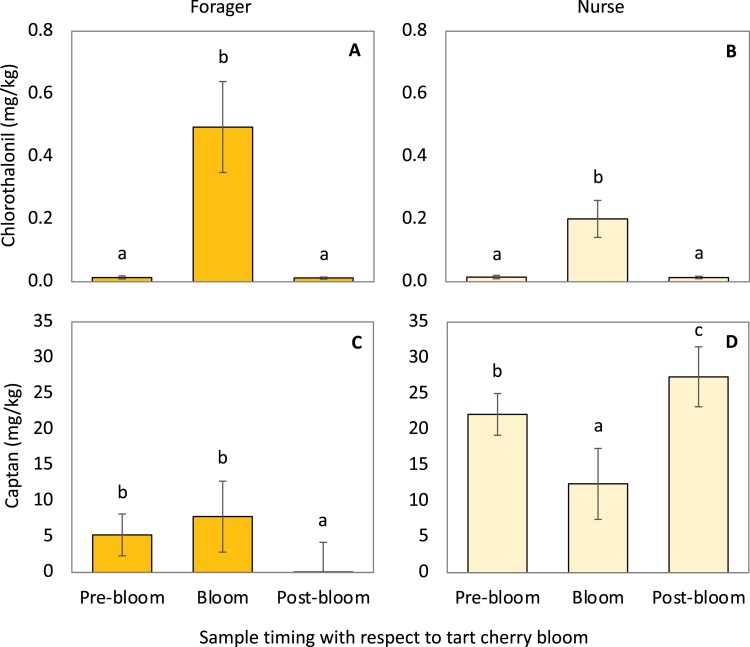
Temporal effects on residue levels in worker bees. Average residues of chlorothalonil or captan detected in foragers (A, C) and nurse bees (B, D) across all three locations and sampling periods related to tart cherry bloom in 2017. Means with different lowercase letters are statistically significant at α = 0.05.

Chlorothalonil residues in both nurse bees and foragers from all sampling locations were significantly higher during tart cherry bloom when compared to the prebloom and postbloom periods (Fig. 3 A and B) (Table 2). Captan residues in foragers were significantly lower posttart cherry bloom compared to the prebloom and bloom time residues (Fig. 3C) (Table 2). Residues of captan in nurse bees were significantly lower during bloom than during both prebloom and postbloom, with the highest residues found during postbloom (Fig. 3D) (Table 2).

#### Pollen and Bee Bread

Chlorothalonil was detected in 86.7% of all the fresh collected pollen samples and in 75% of the bee bread samples, with the highest detections coming from hives at the orchard sites during tart cherry bloom (Table 1). Captan was detected in 100% of all the pollen and bee bread samples with highest detections from hives at the holding yard during tart cherry bloom (Table 1).

#### Wax

Regardless of whether the wax was freshly drawn on drone frames (2016) or from established brood frames (2017), only chlorothalonil was detected in 58.3% of the samples in 2016 and 20.8% of the samples in 2017. All of the detections in wax came from hives at the orchard sites, except for one sample in 2016 that came from the holding yard (Table 1).

### Pollen Composition, Bloom Period, and Fungicide Residues

**Fig. 4.**
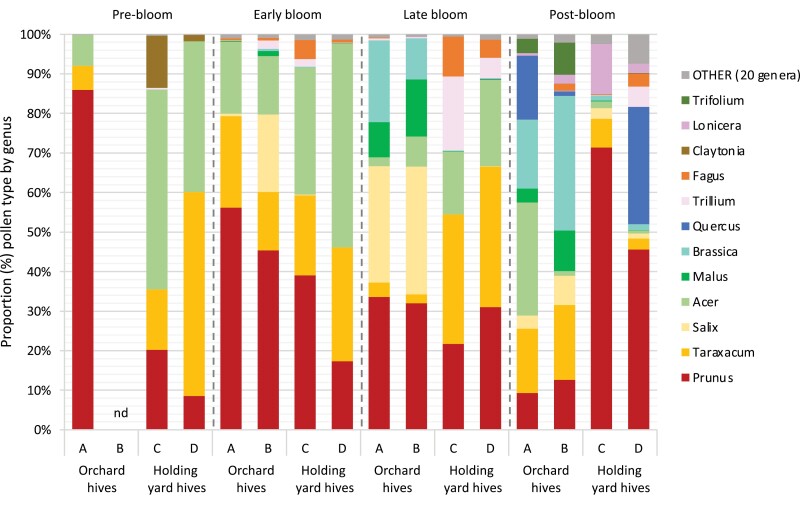
Proportion of pollens, listed by genus, collected by individual hives placed near a tart cherry orchard or a holding yard at four different time periods with respect to tart cherry bloom in 2017. Genera were identified using molecular bar-coding; infrequently collected genera have been lumped together in OTHER. Prebloom samples were collected 9/10 May 2017. Early bloom samples were collected 15/16 May 2017. Late bloom samples were collected 18/19 May 2017. Postbloom samples were collected 31 May 2017. Pollen was collected from a trap placed under each hive. nd = no data due to trap failure.

A total of 32 plant genera were identified among the pollens collected by bees in this study. Of these, the top 12 genera collected in terms of frequency included plants commonly found in and around orchards and adjacent woodlands (Fig. 4). Every sample contained *Prunus* (stone fruit including cherries) and *Taraxacum* (Asteraceae) (dandelion) pollen. Most samples contained *Acer* (Sapindaceae) (maple) pollen. Bees in the orchard hives also collected quantities of *Salix* (Salicaceae) (willow), *Malus* (apple and crabapple), *Brassica* (Brassicaceae) (mustards), and *Trifolium* (Fabaceae) (clovers). Bees in the holding yard hives also collected quantities of *Claytonia* (Montiaceae) and *Trillium* (Melanthiaceae) (woodland wildflowers), and *Fagus* (Fagaceae) (beech). At the postbloom sample period, *Quercus* (Fagaceae) (oak) and *Lonicera* (Caprifoliaceae) (honeysuckle) appeared in the samples.

**Fig. 5.**
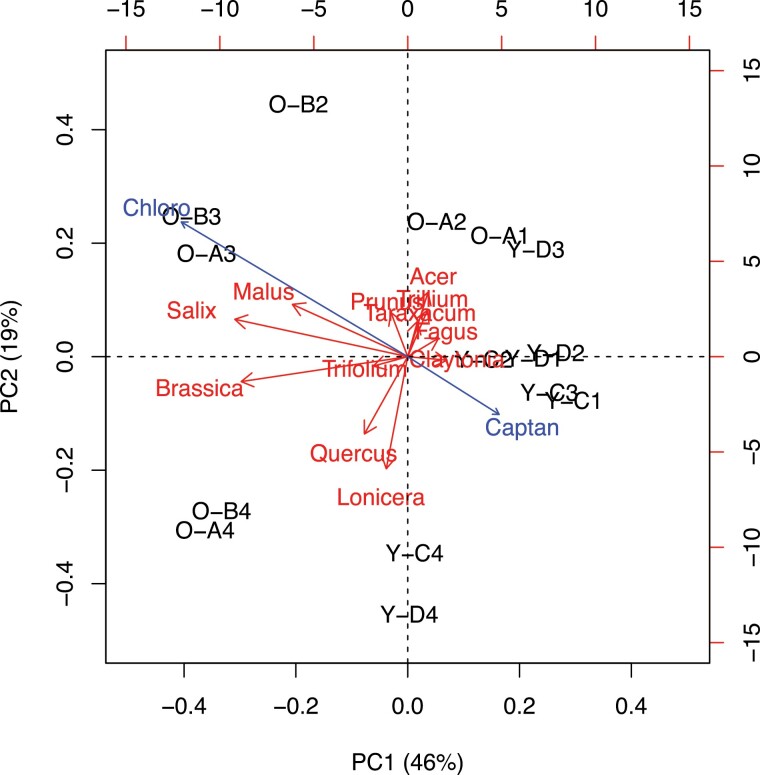
Principal Components Analysis biplot of PC1 and PC2 showing how the top 12 pollen genera collected from 4 hives at 4 time periods reveals two clear gradients. PC1 is a gradient of hives located at an orchard (less than zero) versus a holding yard (greater than zero). PC2 is a gradient of postbloom (less than zero) to all the rest of the bloom periods (greater than zero). Arrows represent vectors pointing to either the 12 pollen genera (*Acer, Brassica, Claytonia, Fagus, Lonicera, Malus, Prunus, Quercus, Salix, Taraxacum, Trifolium,* and *Trillium*) or the two fungicides (Chlorothalonil and Captan). Codes are used to represent location (O = orchard; Y = holding yard), hive (A, B, C, D), and sample time (1 = pre bloom; 2 = early bloom; 3 = late bloom; 4 = post bloom).

Visual inspection of the PCA biplot of PC1 and PC2 showing the top 12 pollen genera collected from 4 hives at 4 time periods suggested two clear gradients (Fig. 5). It appears that chlorothalonil was more prevalent early (PC2) and in orchard samples (PC1), while captan was associated with the later bloom period (PC2) and in holding yard samples (PC1) (Fig. 5). Linear regressions of PC1 scores against fungicide concentrations show that chlorothalonil explains most of the variation in that axis alone (*Adj R*^2^ = 0.569, *F* = 10.24, df = 2,12, *p* = 0.002543) (Table 3). Linear regressions of PC2 scores against bloom period categories show that postbloom was significantly different from early bloom, which was the default reference level of the category and summarized in the model intercept (*Adj R*^2^ = 0.647, *F* = 9.555, df = 3, 11, *p* = 0.002132) (Table 4). Together, these linear regressions quantitatively corroborate our visual (qualitative) interpretations of the PCA biplot. PC1 and PC2 data met the general linear model assumptions of normality (i.e., based on visual inspection of a Q–Q plot as well as results of a Shapiro-Wilk Test, *p* > 0.05), independent observations (Durbin-Watson Test, *p* > 0.05), and linearity and homoscedasticity (*p* > 0.05) of the residuals.

**Table 3.** Parameter estimates for a linear model where PC1 scores comprised the dependent variable and fungicide residues (continuous covariate) comprised the independent variable. *Adj R*^*2*^: 0.569, *F* = 10.24, df = 2,12, *p* = 0.002543

Predictor	Coefficient	Standard Error	*t*	*p*
Intercept	2.28218	1.87515	1.217	0.24697
Chlorothalonil	−0.66495	0.16072	−4.137	0.00138**
Captan	0.06058	0.13653	0.444	0.66515

**Indicates α < 0.01.

**Table 4.** Parameter estimates for a linear model where PC2 scores comprised the dependent variable and bloom period (categorical factor) comprised the independent variable. *Adj R*^*2*^ = 0.647, *F* = 9.555, df = 3,11, *p* = 0.002132

Predictor	Coefficient	Standard Error	*t*	*p*
Intercept (early bloom)	2.0510	0.9172	2.236	0.047012*
Late bloom	−0.3880	1.2971	−0.299	0.770427
Postbloom	−6.1652	1.2971	−4.753	0.000597***
Prebloom	−1.5173	1.4010	−1.083	0.301976

***Indicates α < 0.001, **indicates α < 0.01, *indicates α < 0.05.

## Discussion

Samples from hives rented for tart cherry pollination in this study typically had higher detection frequencies and higher average fungicide residue levels compared to samples collected from hives surrounded mostly by semi-natural areas (i.e., hives placed in holding yards). Chlorothalonil residues were always higher in samples from orchard placed hives compared to holding-yard placed hives in every sample type collected. However, maintaining hives in holding yards while orchards in the region were in bloom did not completely protect honey bees from fungicide exposure, especially when environmental conditions favorable to foliar diseases required more intensive fungicide use, as was the case in 2017. For example, 100% of the nurse bee, pollen, and bee bread samples from holding yard hives in 2017 contained detectible captan residues.

The differences we found between forager and nurse bee residues for chlorothalonil and captan may relate to the different exposure routes, processing, and pharmacokinetics of the two fungicides. Chlorothalonil is lipophilic (water solubility 0.6 mg liter^−1^) whereas captan is 6.3× more water soluble (water solubility 3.8 mg liter^−1^) and is applied to trees as a water-dispersible granule (Bernard and Gordon 2000, Raman 2014). Due to these chemical properties, we would expect chlorothalonil to be detected at higher levels in wax, pollen, and bee bread; whereas captan would be expected to become concentrated in nectar. Nurse bees process pollen into bee bread and consume concentrated nectar within the hive, whereas forager bees often get their food from fresh pollen and nectar while on a foraging trip (Winston 1991). As expected, based on these differences, we observed higher captan residues in nurse bees compared to foragers, as well as higher residues of captan in bee bread compared to chlorothalonil.

The high residue levels we found in our pollen samples compared with the other sample types supports the idea that pollen is a primary source of fungicide exposure in honey bees. The range of chlorothalonil residues in pollen detected in this study is similar to the chlorothalonil residues detected in past studies (Mullin et al. 2010, Pettis et al. 2013, Roszko et al. 2016), providing further evidence that chlorothalonil is a ubiquitous contaminant in pollen. Captan has also been frequently detected in pollen samples in previous studies, with the maximum level detected in pollen from this study (24.4 mg kg^−1^) similar to the maximum captan residues detected in pollen collected from hives next to apple orchards by Kubik et al. 2000 (18.97 mg kg^−1^), but twice as much as reported across multiple cropping systems by Pettis et al. 2013 (13.8 mg kg^−1^). These differences in residue levels are likely a result of differences in management practices among different crop types in each study, and conditions during the season that would increase the need for fungicide applications on the crop. This highlights the influence that crop type and management practices may have on honey bee exposure to fungicides.

The PCA analysis on residues in pollen mirrors the patterns observed in the residue levels found in worker bees. For example, chlorothalonil was more prevalent in pollen and worker bee samples from orchards compared to pollen and worker bee samples from the holding yard hives. Chlorothalonil in worker bees also increased during the cherry bloom period, corresponding to what we observed in pollen samples in the PCA analysis. Captan in pollen was more associated with late and postbloom periods and more prevalent in pollen from the holding yards than the orchard sites, which is consistent with the high levels of captan found in nurse bees in the holding yards during the postbloom period.

A recent review by Rondeau and Raine (2022) found that fungicide residue levels are typically higher in bee bread than they are in honey bee bodies. However, this was not true in our study where the maximum captan residues found in nurse bee and forager bodies were almost twice as high as the maximum captan residues found in bee bread and pollen. Additionally, the maximum chlorothalonil residues detected in foragers was 7 times higher than the maximum chlorothalonil residues detected in bee bread. A potential reason for this disparity may be in our sample collection timing. The maximum residues for foragers mentioned above all occurred during crop bloom, when one would expect fungicide exposure to be highest as they forage in and around crops being protected against disease. Although the bee bread samples were also collected from hive frames during bloom, we cannot know exactly when this bee bread was processed and stored in the hive. There is potential that this bee bread was made from pollen collected and stored before when fungicides were being used in northern Michigan orchards in bloom. Despite this disparity with other studies, we still found bee bread to have a high fungicide residue detection frequency, with captan residues found in 100% of the bee bread samples collected from all locations, and chlorothalonil found in 100% of bee bread samples from orchard locations. This is similar to the findings of McArt et al. (2017) that fungicides accounted for 94% of the pesticide residues detected in fresh bee bread accumulated during apple bloom. The presence of fungicide residues in bee bread is of concern, due to evidence that fungicide contamination can reduce the beneficial fungi found in bee bread (Yoder et al. 2013). However, the direct health implications of this are still understudied.

The absence of residues detected in larvae samples in this study could be attributed to the timing of the sample collection. In this study, brood frames were removed during peak tart cherry bloom, so the fungicides being brought into the hive during bloom may not have been processed fast enough to make it into the developing larvae’s food source before the larvae being collected. However, it may be that due to limitations in the analytical methods available to us for this study, the high LOD/LOQ from these methods prevented us from detecting residues in larvae. Piechowicz et al. (2018) found captan in 100% of honey bee brood samples collected from hives in apple and pear orchards, with residues ranging from 15.7 to 126.5 mg kg^−1^, which is lower than our LOD (Supp Table 1 [online only]), indicating that this fungicide can be detected in developing brood from hives placed near orchards. Pollen and bee bread are mainly used for feeding brood and are therefore both likely sources of exposure for larvae (Rondeau and Raine 2022). Given the high levels we detected in pollen and bee bread in our study, the lack of residues in larvae are very likely under-estimating the actual fungicide accumulation that occurs in developing larvae. This also highlights the need for improved analytical methods (and access to those methods) for the detection of pesticide residues in honey bee matrices with lower residue levels, such as brood and nectar.

Bees wax is known to be a highly contaminated hive matrix, with residues typically higher than those found in honey or bee bodies (Rondeau and Raine 2022). Captan and chlorothalonil have been documented at extremely high levels in wax from previous studies, with captan residues as high as 3.1 mg kg^−1^ and chlorothalonil as high as 53.7 mg kg^−1^ (Mullin et al. 2010, Simon-Delso et al. 2014). The residues detected in this study did not reach levels quite this high, although fungicides were frequently detected in samples from orchard locations. Contamination in wax is concerning for bee health because it can serve as a direct route of contact exposure for larvae while they develop in wax cells (Rondeau and Raine 2022). Wax also typically persists in a hive for many years, leading to a greater potential of accumulation of residues over time (Rondeau and Raine 2022). The total number of pesticide residues present in beeswax has been correlated with poor colony health, and in some cases colony mortality (Traynor et al. 2016).

Fungicide residues detected in this study were enormously variable, even within the same sample types and collection times. Residues were also likely influenced by the timing of applications made by the surrounding growers within the bee’s foraging range. Yearly variability in weather and disease pressure determines the need for growers to make fungicide applications. For example, our study showed higher residue levels of captan and chlorothalonil in nurse bees in 2017 compared with 2016, coinciding with a period of high apple scab and cherry leaf spot infection risk. Disease pressure during each sampling season was determined using disease models on the MSU Enviroweather website (2018).

Flowering crops are not the only plants that can serve as the source of pesticide exposure in honey bees. Fungicide residues have been found on wild flowers growing near crops, although in wildflowers the levels and frequencies of fungicides detected are typically lower than on the adjacent crops (David et al. 2016). The pollen identification in this study revealed that *Prunus*, *Acer, Taraxacum*, *Salix, Brassica*, *Malus*, *Quercus, Trillium, Fagus,* and *Trifolium* are important floral resources for honey bees in the early spring in northern Michigan. Weeds on the orchard floor, such as *Taraxacum* (dandelion), *Brassica* (wild mustards), and *Trifolium* (clover), could be a possible non-crop route of fungicide exposure for honey bees. For instance, we observed a high percentage of dandelion pollen (28%) in samples from the holding yard that also contained high levels of captan (10.2–13.1 mg kg^−1^). These same pollen samples from the holding yard contained 0% *Malus* pollen, suggesting that the captan may have drifted onto dandelions within a nearby orchard, leading to a high level of exposure for the holding yard bees. One practice that orchard growers can adopt to minimize this hazard is to mow or otherwise remove flowering weeds from the orchard floor prior to applying plant protectants. However. a more diverse diet is associated with better health outcomes for honey bee colonies (Huang 2012; Di Pasquale et al. 2013, 2016), so planting or conserving spring blooming plants near orchards is often cited as a way to benefit bees near farms. For these reasons, it is vital when land owners are increasing the number of blooming plants near orchards (May et al. 2015, Isaacs et al. 2017), that they also be utilizing the latest technologies meant to minimize drift onto non-crop plants (e.g., https://sprayers101.com/).

The levels of chlorothalonil detected in this study often exceeded the levels known to cause negative health effects in previous toxicology studies. For example, Pettis et al. (2013) found that *Nosema* infections increased in bees that consumed pollen with high fungicide loads, with chlorothalonil 4.49 mg kg^−1^ residues in pollen coinciding with increased susceptibility to *Nosema* infection and a decreased ability to withstand the infection. The amount of chlorothalonil in 75% of our orchard collected pollen samples exceeded the concentration associated with increased *Nosema* risk. Another study observed a dramatic increase in larval mortality when larvae were reared on a diet containing chlorothalonil at 34 mg kg^−1^ (Zhu et al. 2014). Additionally, food contaminated with 0.01 mg kg^−1^ chlorothalonil (same as our study’s LOD) fed to honey bees in vitro was found to significantly alter the structure and functional potential of the bacterial community within the honey bee gut (Kakumanu et al. 2016). Increases in disease susceptibility and larval mortality, as well as changes to the functional potential of the honey bee bacterial community, can have serious repercussions for bee health. The levels of chlorothalonil detected in our study under current orchard disease management are concerning, given the health effects demonstrated in laboratory studies where honey bees were exposed to similar concentrations.

Captan has also been shown to have negative health implications for developing honey bees. In a laboratory study conducted by Mussen et al. (2004), no larvae completed development to adulthood after being fed a diet containing a concentration of 80 mg kg^−1^ captan. Although the concentration of captan administered in that study was higher than the residue levels of captan we observed in pollen and bee bread samples, the severity of the larval response to captan is highly concerning. Future studies should investigate the effects of more field-relevant fungicide doses on larval development, with special attention to the processing of foods in to larval jelly which may greatly alter the concentrations of containments larvae are exposed to in the real world (Lucchetti et al. 2018). Despite being commonly found in honey bee hives (Kubik et al. 2000, Pettis et al. 2013, Rondeau and Raine 2022), the direct health impacts of captan exposure are still greatly understudied compared to other common fungicides.

The other concern with respect to fungicides and honey bee health is their potential to increase toxicity in combination with other agrochemicals – a phenomenon called ‘synergism.’ A recent review by Siviter and Muth (2020) showed that when bees are exposed to field-realistic levels of multiple agrochemicals there is strong evidence for synergism and increased bee mortality. These synergistic reactions have also been associated with an increased risk of disease and viruses, reduced ability to metabolize food, decreased cellular function, and a higher risk of hypothermia (Glavan and Bozic 2013, Sgolastra et al. 2017). This is an evolving field of study that merits further investigation.

More research is necessary to understand the best methods to balance the need for orchard disease management and the need for healthy bee hives for pollination. We now know the specific exposure rates that bees experience immediately before, during, and after tart cherry bloom in Michigan. Future studies should investigate how honey bee health directly relates to the fungicide accumulation within hives being rented for orchard pollination. Many management recommendations are currently made to growers with the intent to reduce bee fungicide exposures from bloom-time applications; these recommendations include spraying at night when bees are inactive, placing hives in buffered locations (behind tree lines on hill sides), avoiding applications at particular percentages of peak bloom, and carefully calibrating sprayers (May et al. 2015, Isaacs et al. 2017). It would be valuable to quantify the fungicide residues in bee hives under these suggested strategies and compare those levels to the residues found in this study under current standard orchard management practices. Additionally, our study did not investigate these pesticide residues as they related to wild bees. Previous studies have found that orchards can also be sources of pesticide exposure to wild bees due to the high levels of pesticides occurring in pollen, nectar, and soil collected from orchards (Mallinger et al. 2015, Kuivila et al. 2021, Rondeau et al. 2022). Residue accumulation in soil could pose a particular risk to soil-dwelling wild bees residing near orchards, such as in hibernating bumble bee queens (Rondeau et al. 2022).

Our work provides evidence that honey bees will encounter fungicide residues when interacting with spring-blooming tart cherry orchards at levels previously determined to be detrimental to honey bee health. Honey bee hives within the foraging range of orchards in bloom in eastern North America can be exposed to fungicides when they are applied to prevent fungal diseases in the crop. The interaction of hive location, worker role, and the influence of weather on disease pressure can result in variable levels of exposure risks for bees associated with these orchard systems. Honey bees in this study collected a variety of pollens from both wild and cultivated spring blooming plants. Drift onto non-crop flowers is another likely source of fungicide contamination. Recommendations to growers to avoid spraying fungicides when bees are actively foraging, and to mow off flowers on the orchard floor before spraying, may help minimize exposure. While gaps remain in our understanding of how fungicide exposure relates to honey bee colony health at the field level, this study increases our understanding of exposure in honey bees under current bloom time orchard management in this region. Further research is needed to balance necessary crop disease management with necessary pollination services and long-term pollinator health.

## Supplementary Material

toad008_suppl_Supplementary_Figure

toad008_suppl_Supplementary_Materials
