# Supplementary figures and images for: Fungicide Exposure in Honey Bee Hives Varies By Time, Worker Role, and Proximity to Orchards in Spring
Source: J Econ Entomol. 2023 Jan 27;116(2):435–46. doi: 10.1093/jee/toad008 (PMC10148177; doi:10.1093/jee/toad008)

**Scree Plot**

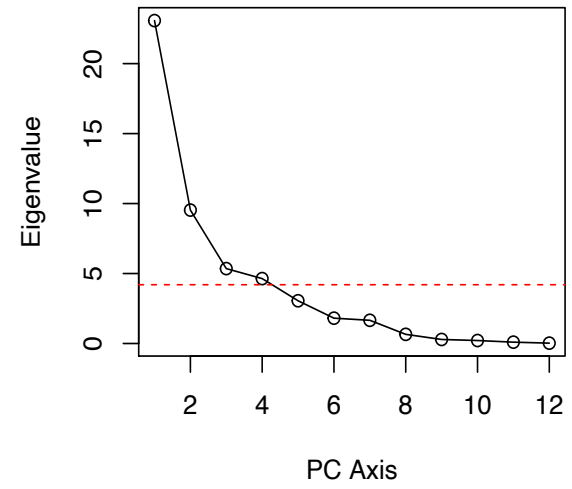

Supplement: toad008_suppl_Supplementary_Figure [file toad008_suppl_supplementary_figure.pdf]
