# Supplementary material for: Fungicide Exposure in Honey Bee Hives Varies By Time, Worker Role, and Proximity to Orchards in Spring
Source: J Econ Entomol. 2023 Jan 27;116(2):435–46. doi: 10.1093/jee/toad008 (PMC10148177; doi:10.1093/jee/toad008)
Supplement: toad008_suppl_Supplementary_Materials [file toad008_suppl_supplementary_materials.docx]

**SUPPLEMENTAL MATERIALS**

**SUPPLEMENTAL TABLES**

**Table S1.** Sampling dates and accumulated growing degree days (GDD) for each year and sampling period of the study. GDD accumulation starting on the first (January 1) of each sampling year, with base 42. Obtained from MSU Enviroweather (using the Baskerville-Emin calculation method at the default setting of base 42°F (5.6°C) beginning January 1.

| **Year** | **Sampling Period** | **Site(s)** | **Sampling Dates** | **GDD** |
| --- | --- | --- | --- | --- |
| 2016* | Pre-Bloom | Holding yard | 9-May | 330 |
|  |  | Orchards | 10-May | 300 |
|  | Bloom | Holding yard | 24-May | 492 |
|  |  | Orchards | 23-May | 466 |
| 2017 | Pre-Bloom | Holding yard | 9-May | 329 |
|  |  | Orchards | 10-May | 337 |
|  | Bloom | Holding yard | 19-May | 463 |
|  |  | Orchards | 19-May | 483 |
|  | Post-Bloom | Holding yard | 31-May | 643 |
|  |  | Orchards | 1-Jun | 656 |
| *2016 post-bloom samples were not able to be collected due to bear damage. | | | | |

**Table S2.** Limit of Quantification (LOQ) and Limit of Detection (LOD) for each fungicide analyzed.

| **Chemical** | **LOQ (mg/kg)** | **LOD (mg/kg)** |
| --- | --- | --- |
| Chlorothalonil | 0.03 | 0.01 |
| Captan | 0.05 | 0.017 |
| Thiophanate-Methyl | 0.53 | 0.16 |

**SUPPLEMENTAL FIGURE LEGEND**

**Figure S1.** Scree plot of eigenvalues used to determine whether a particular principal component (PC) is significant in terms of explaining variation in the multivariate analysis; PC1, PC2, PC3, and PC4 all fall above the red line identifying the average of the eigenvalues, which is the threshold used to determine significance (Kaiser-Guttman test). PC1 (46%) and PC2 (19%) explained the majority of the variability in the model and were selected for examination in the biplot and subsequent linear regressions.
